# Imaging research, diagnosis, and treatment advances of post-stroke cognitive impairment

**DOI:** 10.3389/fnimg.2026.1691870

**Published:** 2026-05-04

**Authors:** Mengyi Huang, Qunbo Jia, Yushu Ouyang, Xiaoteng Feng, Ronghui Ju

**Affiliations:** 1 Department of Radiology, The People’s Hospital of China Medical University, The People’s Hospital of Liaoning Province, Shenyang, Liaoning, China; 2 Graduate School, Dalian Medical University, Dalian, Liaoning, China

**Keywords:** cerebral small vessel disease, imaging, ischemic stroke, multimodal neuroimaging, post-stroke cognitive impairment

## Abstract

Post-stroke cognitive impairment (PSCI) has garnered widespread attention due to its high incidence and its association with increased risk of stroke recurrence and mortality. Growing evidence indicates that early prediction of PSCI and the implementation of effective interventions can help delay disease progression and improve long-term patient outcomes. With advances in imaging technology, the role of neuroimaging has evolved from traditional anatomical localization to a multimodal assessment system that integrates macrostructural, microstructural connectivity, and molecular metabolic information. Imaging features can serve as objective and reproducible quantitative indicators, sensitively capturing subtle pathological changes in brain tissue, thereby providing a reliable basis for clinical diagnosis, treatment strategy formulation, and prevention. This review systematically summarizes recent research progress in the clinical diagnosis and imaging characteristics of PSCI. It focuses on analyzing the impact and underlying mechanisms of specific biomarkers, gene expression, cerebral small vessel disease, and cerebral perfusion abnormalities on cognitive function, and further explores the application prospects of advanced imaging technologies in the assessment of PSCI.

## 1 Introduction

Stroke is a neurological disease with a high incidence worldwide; in China, the incidence, prevalence, and mortality of stroke are particularly high (Tu and Wang, 2023). Acute ischemic stroke (AIS) accounts for approximately 60 to 80% of overall stroke incidence, and its incidence is on the rise. With the continuous aging of the population, the clear trend of stroke recurrence, and the poor control of risk factors such as hypertension, the prevention and treatment of stroke are currently facing great pressure and challenges (GBD 2019 Stroke Collaborators, 2021; Thayabaranathan et al., 2022).

Post-stroke cognitive impairment (PSCI) refers to objectively confirmed cognitive deficits emerging after stroke that persist beyond the acute phase and are typically reassessed at 3–6 months, with persistence at 6 months considered a key diagnostic/evaluative benchmark, ranging from mild impairment to post-stroke dementia. The global incidence of PSCI is approximately 20 to 80%, and up to 90% of patients experience acute-phase cognitive impairment (Tsiakiri et al., 2024; Aben et al., 2021), and approximately 60% of survivors of stroke in the first year after stroke (El Husseini et al., 2023; Ai et al., 2025). As a common complication of stroke, the main clinical manifestations of PSCI are varying degrees of impairment in attention, memory, visuospatial ability, language ability, and executive ability. These are sometimes accompanied by mental and behavioral abnormalities, which often lead to a substantial reduction in the ability of a patient to learn and work, as well as to perform daily life and social interactions (He et al., 2023). If patients with PSCI do not receive effective interventions, approximately one-third of them will go on to develop severe dementia or even disability. In addition, although the risk of recurrent stroke and death associated with cognitive impairment after stroke has been reported inconsistently in studies over the past three decades, a recent meta-analysis of 27 studies (*n* = 39,412 patients) showed that cognitive impairment after stroke was associated with a 59% increased risk of recurrent stroke and a 2-fold increased mortality (Dowling et al., 2024). The early identification and prediction of cognitive impairment prognosis in patients with AIS is critical.

Advances in neuroimaging technology have enabled radiological features to directly visualize the structural etiology of cognitive impairment, while quantitative indices provide the sensitivity required to detect subtle pathological alterations in brain tissue. Consequently, imaging has become a cornerstone for the early detection, mechanistic elucidation, and clinical decision-making in PSCI. This paper reviews the latest progress in the diagnosis and management of PSCI, with a specific focus on the application of radiological characteristics and multimodal imaging techniques.

## 2 Clinical evaluation and risk factors

### 2.1 Neuropsychological assessments

The 2023 American Heart Association (AHA) scientific statement on post-stroke cognitive impairment clearly recommends simple cognitive screening and assessment for stroke patients (El Husseini et al., 2023). The most common cognitive assessment tools are the Mini-Mental State Examination (MMSE) and the Montreal Cognitive Assessment (MoCA). Of these, the MMSE is relatively simple and easy to administer but has limitations such as age and education (Nys et al., 2005; Pendlebury et al., 2010), whereas the MoCA is more reliable, valid, and sensitive (Burton and Tyson, 2015; Gallucci et al., 2024). Since post-stroke cognitive changes constitute a dynamic process, assessment results exhibit significant heterogeneity. It is recommended to perform initial screening or testing during the acute phase of stroke or prior to hospital discharge, followed by a second assessment at 3–6 months after stroke (Hwang and Fick, 2025).

In addition to direct patient assessments, informant-based evaluations, such as the Informant Questionnaire on Cognitive Decline in the Elderly (IQCODE), play a crucial role in the comprehensive cognitive assessment of stroke survivors, particularly when patients are difficult to assess due to severe motor deficits, aphasia, or reduced consciousness. In the acute phase of stroke, the IQCODE is uniquely valuable for distinguishing pre-existing dementia from the acute decline in cognitive function attributable to the acute stroke event. A higher pre-stroke IQCODE score suggests diminished cognitive reserve and is an independent risk factor for the occurrence and progression of PSCI (Zhao et al., 2021). During the 3- to 6 month follow-up period, the IQCODE can assist in the diagnosis of PSCI, demonstrating a sensitivity of 81% and a specificity of 83%, which is comparable to the diagnostic performance of the MoCA (McGovern et al., 2016). IQCODE scores may also predict future dementia risk, although with relatively low sensitivity (Burton et al., 2021). Given that IQCODE results are less influenced by a patient’s educational level or language deficits, it serves as an excellent complement to scales like the MoCA. Combining the IQCODE with instruments such as the MoCA facilitates a more comprehensive and accurate identification of PSCI, particularly in populations with lower educational attainment or diverse cultural backgrounds.

### 2.2 Risk factors and biomarkers

Genes and certain biomarkers present in blood and cerebrospinal fluid can also assist in the diagnosis of PSCI. Brain-derived neurotrophic factor, protein S100β, soluble intercellular adhesion molecule-1, oxidized low-density lipoprotein, and soluble CD40 ligand are of significant value for the early prediction of PSCI in patients not receiving thrombolytic therapy (Chen et al., 2023; Wang et al., 2023; Gao et al., 2023; Chang et al., 2024). In addition, certain inflammatory markers such as C-reactive protein, erythrocyte sedimentation rate, interleukins, and tumor necrosis factor-*α* may also have predictive value for PSCI, further explaining the potential role of anti-inflammatory interventions in improving cognitive function (Zhang et al., 2022; Tack et al., 2025).

In terms of genetics, the apolipoprotein E4 (*APOE4*) allele is a strong risk factor for Alzheimer’s disease (Koutsodendris et al., 2022; Raven et al., 2017), it significantly increases the accumulation of Aβ peptide and plaque formation, promotes the aggregation of tau proteins, and leads to the generation of neuroinflammation (Yamazaki et al., 2016), with subsequent aberrant changes in neuronal structure and function. Interestingly, Ma et al. (2024) reported that the *APOE4* gene is an independent risk factor for PSCI in AIS patients. APOE4 was a predictor of longitudinal Aβ accumulation (Lim and Mormino, 2017), the interaction between APOE4 and Aβ that increases tau aggregation (Therriault et al., 2021; Kang et al., 2023). Furthermore, a longitudinal FDG-PET study revealed that patients with MCI and APOE4 carriers exhibit reduced regional glucose metabolism in the brain. This suggests that APOE4 affects cognitive function by influencing glucose metabolism (Paranjpe et al., 2019). Additionally, APOE4 is associated with an inflammatory state, as APOE4 carriers have higher levels of inflammatory markers, such as tumor necrosis factor *α* (TNFα) and interleukin 6 (IL-6), in their plasma (Gale et al., 2014).

Other risk factors for PSCI include age, education level, hypertension, diabetes, atrial fibrillation, history of stroke, and hyperhomocysteinemia (Filler et al., 2024; Lu et al., 2019; Ji et al., 2023). Although most studies have concluded that there is no significant association between sex and PSCI, it has been suggested that women have increased risk for PSCI (Yang et al., 2017), which may be related to baseline cognitive reserves or estrogen levels in the body, but the heterogeneity is large and requires further study.

## 3 Neuroimaging predictors

Neuroimaging serves as a critical tool for the early prediction and diagnosis of PSCI. Specific infarct location, global or medial temporal lobe atrophy, and white matter lesions have all been identified as key correlates of PSCI. Furthermore, neurodegenerative changes and cerebral small vessel disease (CSVD) associated with cognitive decline frequently coexist and interact. Utilizing a total CSVD score to evaluate cumulative burden allows for a more comprehensive assessment of the heterogeneity and synergistic effects of vascular pathology.

### 3.1 Strategic infarct location & infarct volume

The occurrence of PSCI is related to the location of the cerebral infarction and the size of the lesion. The location of the lesion is a key determinant of PSCI, particularly the lesion accumulates cortical or associative fibers associated with cognitive function; so when the lesion is located in the paraventricular white matter, basal ganglia, cortex, and/or the dominant hemisphere, the incidence of PSCI is higher, which may be related to asymmetry in the innervation of cognitive and memory functions in the brain. Weaver et al. (2021) concluded that infarcts in the left frontotemporal lobe, left thalamus, and right parietal lobe are strongly associated with PSCI, infarcts in the right occipital lobe, brain stem and cerebellum conveyed a lower risk of PSCI. Furthermore, language and verbal memory impairment were mainly lateralized to the left hemisphere, while impairment of visuospatial functions was associated with right parietal infarcts, which is consistent with Moore’s findings (Moore and Demeyere, 2022). Additionally, hippocampal damage may lead to persistent memory impairments, and there are differences in cognitive alterations after bilateral hippocampal damage (Szabo et al., 2009; Kumral et al., 2015; Blum et al., 2012). The cerebral cortex is mainly responsible for the higher nervous activities of the brain, which are related to cognitive and emotional responses and learning functions (Hénon et al., 2001). The strategic infarction locations of PSCI are representative of different cognitive domains, indicating that these strategic infarction locations are indeed broadly predictive of PSCI. However, when using lesion-symptom mapping to determine the neuroanatomical correlation of cognitive deficits, it is important to consider confounding factors such as interdependence between infarction locations, clinical history, and other brain injuries (e.g., white matter hyperintensities, previous infarctions).

Infarct volume is a significant predictor of clinical outcomes in ischemic stroke, yet the relationship is nonlinear: the association between small infarct volume and prognosis remains unclear, while larger infarct volumes correlate with lower probabilities of favorable functional outcomes (Xu et al., 2025). Multivariate analysis indicates that infarct volume ≥0.054 mL is an independent risk factor for PSCI (Prodjohardjono et al., 2020). Furthermore, larger infarct volumes correlate with increased likelihood of involvement in strategically important regions (e.g., left frontotemporal lobe, left thalamus, right parietal lobe). The location and volume of infarcts synergistically influence cognitive outcomes (Xu et al., 2025; Ospel et al., 2021).

### 3.2 Cerebral atrophy

Cerebral atrophy is mainly characterized by widened sulci, shallow gyri, and enlarged ventricles and subarachnoid space on imaging. Previous studies (Lee et al., 2023; Ball et al., 2022; Mehrabian et al., 2015) have confirmed that brain atrophy is closely related to cognitive impairment, of which medial temporal lobe atrophy has received the most research attention. This may be related to hippocampal atrophy or tau protein and amyloid precursor protein deposition caused by prolonged hypoperfusion (Visser et al., 2023; Takahashi et al., 2019). Godin et al. (2010) reported that the degree of medial temporal lobe atrophy is correlated with PSCI; both a larger white matter lesion (WML) volume and a smaller hippocampal volume significantly increases the risk of severe cognitive impairment and the rate of cognitive decline, with a cumulative effect. A related systematic analysis (Casolla et al., 2019) concluded that the most consistent predictors of cognitive impairment after stroke are overall atrophy and medial temporal lobe atrophy. Furthermore, there is also reportedly a relationship between cortical atrophy and domain-specific cognitive recovery after stroke (Kandiah et al., 2016; Hobden et al., 2023), in the acute/subacute phase after stroke, cortical atrophy and cerebral white matter lesions do not correlate with either generalized or domain-specific PSCI, whereas in the chronic phase, individuals with more severe cortical atrophy (i.e., higher total global cortical atrophy scores) have a higher overall PSCI level than those with less severe cortical atrophy. Memory and attention deficits in such patients are more likely to persist into the chronic phase. Takahashi et al. also used MRA source images to semi-quantitatively evaluate medial temporal lobe atrophy (MTLA) and studied and confirmed its correlation with PSCI (Takahashi et al., 2019). Therefore, incorporating the medial temporal lobe atrophy (MTA) scale into routine stroke MRI evaluation will help clinicians pay attention to the development of stroke patients.

### 3.3 CSVD burden and markers

#### 3.3.1 White matter lesions

Cerebral white matter lesions are focal, diffuse, or even large fused foci of high signal in the white matter regions of the cerebral hemispheres on T2-weighted imaging. It is currently presumed that cerebral white matter lesions are the result of long-term cerebrovascular lesions leading to the hardening of small blood vessels in the brain, which causes demyelination and gliosis. Cerebral white matter demyelination lesions can significantly affect the development of cognitive dysfunction (Frantellizzi et al., 2020). This may be related to the finding that cerebral white matter lesions affect the neural network system and destabilize nerve fiber conduction (Han et al., 2018). Maintaining the integrity of the brain’s white matter ensures normal neural transmission. The white matter tracts connect different brain regions, forming specialized pathways. As the extent of white matter lesions expands and more transmission fibers are damaged, patients’ cognitive functions will gradually decline. Moreover, these lesions can affect the energy metabolism and related functions of the cerebral cortex, leading to functional impairment. Although patients with cerebral white matter lesions are clinically unremarkable in the early disease stages, progression tends to be rapid. Verdelho et al. (2010) followed up 639 patients with cerebral white matter lesions over a period of 3 years and revealed that 90 patients progressively evolved into cases of dementia, whereas 147 progressively evolved into cognitive dysfunction without dementia. Another study (Zamboni et al., 2017) demonstrated that white matter lesions, which are a risk factor for PSCI, are independently associated with MoCA scores, and frontal white matter high signal accelerates the development of post-stroke dementia. With increased severity of periventricular white matter hyperintensities (WMH) and deep white matter hyperintensities, visual learning, verbal learning and recall were significantly impaired (Hilal et al., 2021). The study by Coenen et al. (2024) also identified the left anterior thalamic radiation and the forceps major as strategic white matter tracts associated with specific domain cognitive functions in stroke patients. The volume of WMH in the left anterior thalamic radiation was significantly associated with attention, executive function cognitive performance, and information processing speed, while the volume of WMH in the forceps major was significantly associated with information processing speed.

#### 3.3.2 Cerebral microbleeds

The anatomical distribution of cerebral microbleeds (CMBs) often varies depending on the underlying etiology. In cerebral amyloid angiopathy (CAA), CMBs are predominantly located in the cortical gray matter and juxtacortical white matter, whereas in hypertensive arteriolosclerosis, they are more commonly distributed in deep brain structures (e.g., deep gray matter, white matter). This pathophysiological pattern can be explained by *β*-amyloid accumulation leading to vascular wall thickening, remodeling, and blood–brain barrier dysfunction. Studies have shown that strictly lobar CMBs or cortical superficial siderosis (cSS) are significantly associated with Aβ deposition (with odds of elevated Aβ being more than 4 times higher compared to patients without CMBs) (Okine et al., 2023). However, research by Zheng et al. (2022) indicated that cognitive function is significantly negatively correlated with the number of CMBs, but not with their location. Based on existing literature, we propose several potential mechanisms by which CMBs may contribute to cognitive impairment: (1) CMBs may serve not only as markers of local vascular injury but also of generalized vasculopathy and neurodegenerative damage (Akoudad et al., 2016); (2) Elevated serum levels of Aβ1-42 and pTau-181 in patients with CMBs suggest possible co-existence with Alzheimer’s pathology (Poliakova et al., 2016); (3) CMBs may reflect impaired blood–brain barrier integrity, leading to impaired clearance of toxic substances such as A*β* (Gold et al., 2021); (4) CMBs frequently coexist with WMH, jointly contributing to disruption of brain network connectivity (Choe et al., 2025). Nonetheless, some studies have found no association between CMBs and an increased risk of dementia (Pinheiro et al., 2025).

#### 3.3.3 Cortical superficial siderosis

CSS represents deposition of hemosiderin within the leptomeningeal/superficial cortical macrophages, indicative of prior microscopic subarachnoid hemorrhage or vascular leakage. cSS is regarded as a hallmark feature of CAA and may predispose to intracerebral hemorrhage and dementia (Umino et al., 2021). Studies suggest that CAA-associated cSS may impair brain function via the cytotoxic effects of blood breakdown products (Durrani et al., 2023). In addition, iron-mediated cytotoxicity, reactive astrogliosis, and inflammatory responses can further provoke secondary injury to adjacent cortex and vessels, leading to recurrent hemorrhage and cognitive decline (Charidimou et al., 2020; Auger et al., 2023). In patients with CAA, the presence of cSS is associated with reduced processing speed; together, increased peak width of skeletonized mean diffusivity (PSMD), impaired cerebrovascular reactivity (CVR), and brain atrophy account for roughly half of CAA’s effect on cognition (Durrani et al., 2023).

#### 3.3.4 Perivascular spaces

Perivascular spaces (PVS) are interstitial fluid channels surrounding cerebral blood vessels, constituting part of the neurovascular unit and the blood–brain barrier, and serving as pathways for the clearance of metabolites such as β-amyloid. PVS visible on MRI can be quantified and may reflect underlying CSVD and dysfunction of the metabolic clearance pathway (i.e., glymphatic dysfunction) (Wardlaw et al., 2020). Cohort studies have shown that PVS burden is associated with an increased risk of all-cause dementia and Alzheimer’s disease, with risk rising as PVS burden increases, independent of vascular risk factors and prevalent cardiovascular disease (Romero et al., 2022). Furthermore, higher PVS burden in the centrum semiovale (CSO) region has been linked to an elevated risk of mild cognitive impairment (MCI) (Pase et al., 2023).

#### 3.3.5 Lacunar infarction

Lacunar infarcts, resulting from occlusion of a single penetrating artery, are a hallmark of CSVD (Maksimova and Gulevskaya, 2019) and contribute to PSCI through two interconnected mechanisms: strategic location and cumulative burden. The cognitive impact depends critically on infarct location; lesions disrupting key thalamocortical circuits--particularly the anterior thalamic nuclei (ATN), a central node in the Papez circuit--can cause disproportionate deficits. A mouse model of selective ATN infarction demonstrated significant spatial learning and memory impairments with reduced neural connectivity from the retrosplenial cortex to the thalamus, exemplifying a “disconnection syndrome” where a small strategic infarct disrupts specific neural circuits essential for cognition without causing large-scale cortical damage (Catani and Ffytche, 2005; Zhang et al., 2023). While single strategic infarcts are impactful, the cumulative effect of multiple lacunes—termed the “lacunar state”—drives widespread vascular cognitive impairment by disrupting fronto-subcortical networks, manifesting as deficits in executive function and processing speed (Maksimova and Gulevskaya, 2019). Critically, lacunes rarely occur in isolation; they frequently coexist with white matter hyperintensities and cerebral microbleeds, and this synergistic combination amplifies network disruption, accelerating cognitive decline and increasing dementia risk (Nakamori et al., 2020; Yu et al., 2019; Auriat et al., 2019; Wang et al., 2017). Thus, lacunar infarcts serve as robust markers of advanced microangiopathy, contributing to PSCI through both focal disconnection and diffuse network dysfunction.

While individual CSVD markers offer valuable insights, they often coexist and exert synergistic effects on cognitive function. To capture the cumulative burden of CSVD, the concept of a “total CSVD score” has been developed and widely adopted following the STRIVE criteria. The total CSVD score integrates four key MRI markers—WMH, lacunes, CMBs, and PVS—into a pragmatic 0–4 scale (Staals et al., 2014). One point is awarded for moderate-to-severe WMH, ≥1 lacune, ≥1 CMB, and moderate-to-severe basal ganglia PVS (Staals et al., 2015). A higher total burden is independently associated with poorer global cognitive function, particularly in attention and processing speed (Hosoya et al., 2023). Furthermore, severe burden (score >2) predicts long-term cognitive decline and incident dementia in older adults, underscoring its value for risk stratification (Hua et al., 2023; Zhang X. et al., 2025). Thus, the total CSVD score effectively translates heterogeneous imaging findings into a unified measure of cumulative vascular brain injury.

### 3.4 Cerebral perfusion

Chronic hypoperfusion following stroke is a key mechanism underlying cognitive decline, even a slight impairment of cerebral blood flow regulation can significantly impact brain function (Zhang et al., 2017). This may be due to reduced blood flow and perfusion to brain tissue after a stroke, leading to decreased neuronal excitability and a lower metabolic rate. Additionally, ischemia and hypoxia of neurons, combined with cerebrovascular diseases in patients with hypertension and diabetes, can directly damage nerve fibers, further exacerbating cognitive impairment. Notably, inadequate cerebral perfusion plays a role in cognitive dysfunction independently of amyloid, tau protein, and vascular risk factors (Visser et al., 2023; Meng et al., 2023). Sun et al. (2016) reported that cognitive deficits are observed in patients with subcortical ischemic cerebrovascular disease, and that brain perfusion deficits in the temporal frontal lobe, hippocampus, thalamus, and other regions correlate with the degree of cognitive dysfunction. Perfusion imaging can quantify the dynamic changes in cerebral blood flow after stroke. The volume of the ischemic penumbra, the hypoperfusion intensity ratio (HIR, defined as the volume with Tmax >10 s divided by the volume with Tmax >6 s), and collateral circulation status are closely associated with final infarct volume and indirectly influence cognitive function. Meng et al. (2019) stated that, in patients with asymptomatic middle cerebral artery stenosis, poor collateral circulation is often comorbid with cognitive domain impairments. By contrast, patients with good collateral circulation are at relatively low risk of cognitive impairment. Thus, we can evaluate collateral circulation status and long-term prognosis (including PSCI) through CT or MR perfusion imaging.

**Figure 1:**
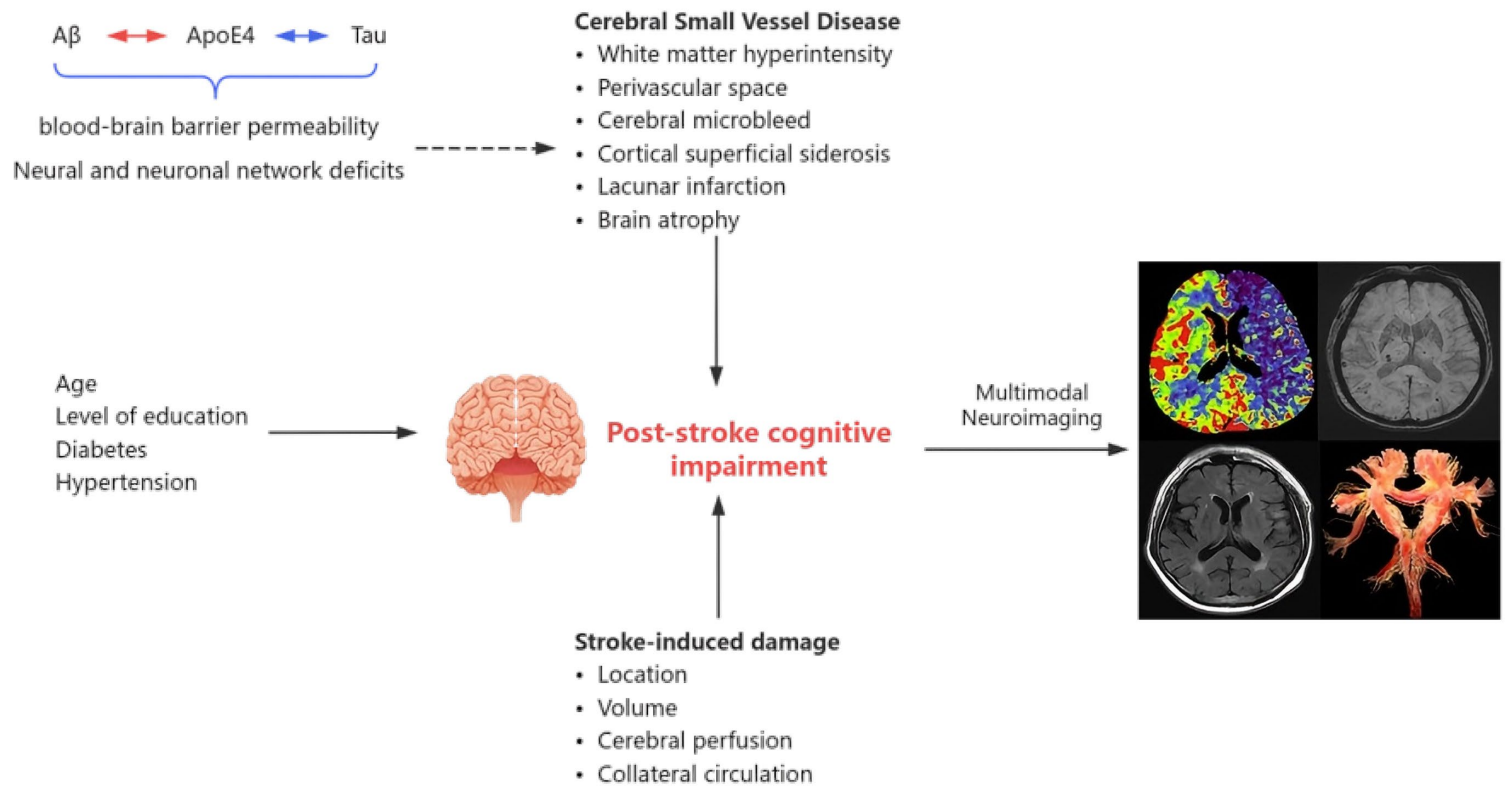
Influencing factors of post-stroke cognitive impairment.

However, it has been suggested (Kalaria et al., 2016) that when the middle cerebral artery is stenosed or occluded, the posterior cerebral artery becomes “lateralized,” resulting in reduced blood flow to its own blood-supplying regions and the dysfunction of structures such as the hippocampus and thalamus. This can affect a variety of cognitive functions, including memory. It has also been reported (Steiner et al., 2021) that imbalances in perfusion between the cerebral hemispheres may contribute to the continued deterioration of cognitive function. Additionally, in a 12-year longitudinal study (Ghaznawi et al., 2021), cerebral blood flow was revealed to be associated with the progression of cerebral atrophy, thereby further supporting the role of cerebral blood flow in neurodegenerative pathologies. Figure 1 summarizes the influencing factors of PSCI.

## 4 The role of multimodal neuroimaging

With advances in imaging technology, the role of neuroimaging in PSCI diagnosis has evolved beyond mere anatomical localization to a multimodal assessment system integrating macroscopic structures, microscopic connectivity, and molecular metabolism. Imaging can visually reveal structural causes of cognitive impairment, such as the location and volume of infarcts, as well as the burden of CSVD. Imaging features—including white matter hyperintensity volume, hippocampal volume, and diffusion tensor imaging (DTI) parameters—serve as objective, reproducible quantitative indicators for disease severity assessment and clinical monitoring. Furthermore, integrating multimodal imaging with radiomics and machine learning holds promise for early risk stratification, individualized etiological differentiation, and treatment decision support in PSCI. This approach may enhance early diagnostic sensitivity, identify reversible pathologies, and optimize rehabilitation and secondary prevention strategies. Table 1 summarizes the applications, advantages, and limitations of multimodal imaging in PSCI.

**Table 1:** The utility, advantages and limitations of multimodal imaging in PSCI.

Imaging modality	Specific technique	Application in PSCI and advantages	Limitations
CT imaging	Non-contrast CT	Acute phase: exclude intracranial hemorrhage, detect large infarcts; assess cerebral atrophy.	Low sensitivity for early ischemic changes and subtle pathologies.
	CT angiography (CTA)	Visualize the culprit vessel, guide endovascular therapy.	Requires iodinated contrast; risks of radiation and allergic reaction.
	CT perfusion (CTP)	Assess ischemic brain tissue, identify ischemic penumbra.	Higher radiation dose; quantitative accuracy influenced by multiple factors.
MRI	Conventional MRI (T1/T2/FLAIR, etc.)	Precise detection of small vessel disease markers and characteristic atrophy (e.g., hippocampal).	Longer scan time, contraindicated for certain metal implants, higher cost.
	Perfusion-weighted imaging (PWI)/arterial spin labeling (ASL)	Quantitative assessment of focal/diffuse cerebral hypoperfusion; identify penumbra or chronic hypoperfusion regions.	PWI requires contrast agent; ASL has lower signal-to-noise ratio, motion-sensitive.
	Diffusion tensor imaging (DTI)	Assess impairment of white matter tract integrity in lesion areas, indicating involvement of cognition-related pathways	Results influenced by local WM microstructure, crossing fibers, free water effects.
	DTI-ALPS	Indirect, non-invasive assessment of glymphatic system function; studies link to edema dynamics, neuroinflammation, and cognitive outcomes.	Index values confounded by factors like WM microstructure; requires multimodal interpretation with other metrics (e.g., PSMD, MD).
	Functional MRI (fMRI)	Assess brain functional connectivity and hemodynamic changes; reveal association between network reorganization and cognitive impairment.	Sensitive to motion and electromagnetic interference; data prone to artifacts;
Other functional techniques	Functional near-infrared spectroscopy (fNIRS)	Monitor cortical activation (e.g., prefrontal); assess PSCI severity and stratification.	Limited penetration depth (mainly cortical); spatial resolution lower than fMRI.

In the acute phase, CT is primarily utilized to exclude intracranial hemorrhage and detect large infarcts, while also providing preliminary information on cerebral atrophy and white matter lesions. However, its sensitivity for early ischemic changes and subtle pathologies remains limited. CT angiography (CTA) and CT perfusion (CTP) can visualize the culprit vessel and ischemic brain tissue, offering valuable guidance for endovascular therapy. MRI, on the other hand, allows precise detection of small vessel disease markers and characteristic atrophy patterns (e.g., hippocampal atrophy). Perfusion-weighted imaging (PWI) and arterial spin labeling (ASL) enable quantitative assessment of focal or diffuse cerebral hypoperfusion, identifying regions of ischemic penumbra or hemodynamic compensation due to chronic hypoperfusion, thereby aiding in evaluating potential benefits from reperfusion or hemodynamic interventions (Mijajlović et al., 2017).

DTI can capture impairments in the integrity of white matter fiber tracts within lesion areas, indicating involvement of pathways associated with cognitive function. Diffusion Tensor Imaging Analysis along the Perivascular Space (DTI-ALPS) provides a rapid, noninvasive, and contrast-free method for indirectly assessing the function of the brain’s glymphatic system. Multimodal studies link glymphatic dysfunction with cerebral edema dynamics and functional outcomes, suggesting that perivascular transport failure may participate in the cascade from cytotoxic-vasogenic edema to secondary neuronal injury and cognitive sequelae. This offers a novel perspective for understanding potential mechanisms of PSCI, such as impaired edema clearance, neuroinflammation, and accumulation of metabolic waste (Grigoras et al., 2025; Sun et al., 2025). Relevant studies have shown that the ALPS index on the lesion side in patients with ischemic stroke is significantly lower than in healthy controls, with the most pronounced reduction occurring in the acute phase, followed by partial recovery over weeks to months. This finding provides a reliable reference for effectively distinguishing early cognitive impairment and predicting unfavorable outcomes at 6 months. Furthermore, the ALPS index is associated with cognitive function and disrupted structural network connectivity in patients with vascular cognitive impairment (VCI). Global topological metrics of the structural network (e.g., global efficiency, Eg) mediate the relationship between glymphatic function and cognition (Song et al., 2022). However, ALPS values may be influenced by factors such as local white matter microstructure, crossing fibers, and free water effects, necessitating multimodal interpretation in combination with other diffusion metrics (e.g., PSMD, MD).

Functional magnetic resonance imaging (fMRI) is primarily used to assess brain functional connectivity and hemodynamic changes, revealing the association between network reorganization and impairment in cognitive domains (Han et al., 2025). Combined analysis based on ASL-fMRI has demonstrated significant correlations among neurovascular coupling (NVC), cognitive scores, and white matter lesion burden in patients with subcortical vascular cognitive impairment (SVCI) and PSCI (Ruan et al., 2023). Utilizing resting-state fMRI, Rao et al. (2022) evaluated different states of dynamic functional network connectivity and their topological network properties, finding that brain networks in PSCI patients tend to shift toward a “suboptimal state” with incomplete compensation centered on the frontoparietal network. However, fMRI has limited resistance to motion and electromagnetic interference, and data quality is susceptible to artifacts. In contrast, functional near-infrared spectroscopy (fNIRS) offers advantages such as high temporal resolution, portability, and motion robustness, effectively compensating for the limitations of fMRI in monitoring stroke populations (Ai et al., 2025). fNIRS also shows considerable potential in clinical research. Studies have revealed significantly reduced activation in the left dorsolateral prefrontal cortex (L-DPFC) in PSCI patients, and its activation features--particularly L-DPFC centroid values--can serve as sensitive indicators for assessing PSCI severity. Integrating fNIRS features with clinical variables enables effective stratification of PSCI severity (Zhang H. et al., 2025).

Other neurophysiological techniques, such as electroencephalography (EEG) and magnetoencephalography (MEG), can serve as supplementary tools to traditional cognitive assessments. They offer strong objectivity, greater sensitivity to subtle changes in brain functional states, and detect abnormalities earlier than behavioral manifestations (e.g., significantly reduced peak alpha frequency in PSCI patients) (Zhao et al., 2025).

Beyond providing qualitative insights into structural and functional abnormalities, multimodal imaging enables quantitative assessment of PSCI predictors through automated software and deep learning algorithms. Tools such as FreeSurfer, FSL, and SPM are widely used for automated volumetry of cortical thickness, hippocampal volume, grey matter, and intracranial volume, offering reproducible measurements essential for large-scale studies (Fischl, 2012). For lesion-specific quantification, convolutional neural networks (CNNs)—particularly U-Net architectures—have demonstrated high accuracy in segmenting white matter hyperintensities from FLAIR images across multi-center datasets (Shan et al., 2021; Sundaresan et al., 2021). Similarly, 3D U-Net models enable robust detection of cerebral microbleeds on T2*GRE and SWI sequences, achieving performance comparable to expert raters (Tsuchida et al., 2024). Deep learning has also been applied to grade enlarged perivascular spaces, facilitating automated risk stratification (Williamson et al., 2022). These AI-based approaches minimize inter-rater variability, reduce manual effort, and allow seamless integration of total CSVD burden scores into clinical workflows, ultimately enhancing the prediction of post-stroke cognitive decline (Zhang X. et al., 2025).

## 5 Management and intervention of PSCI

There is no single cure for PSCI, so management is multifaceted–addressing vascular risk factors, cognitive symptoms, and rehabilitation. Studying the factors that influence PSCI may help with the development of targeted treatments and the formulation of personalized prevention and treatment plans. Therefore, controlling the risk factors of stroke and PSCI, such as the reasonable control of hypertension, diabetes mellitus, and hyperlipidemia, can prevent and reduce the occurrence of PSCI. However, there are currently no specific approved drugs for treating PSCI; instead, commonly used medications often draw from AD treatment protocols (El Husseini et al., 2023). Drugs that are widely used clinically to treat patients with PSCI include the cholinesterase inhibitors Donepezil and Carbazochrome. In addition, calcium antagonists (e.g., Nimodipine), excitatory amino acid receptor blockers (e.g., Memantine) (Kim et al., 2020), and some traditional Chinese medicines (e.g., Chuanxiong Rhizoma, Acorita Tatarinowii Rhizoma, Pheretima) have achieved good efficacy in the treatment of PSCI (Shen et al., 2022; Pei et al., 2020). But, the evidence for traditional Chinese medicine is limited. Most studies are small-scale or regional cohorts, which need to be verified by large samples. Moreover, the intervention is mainly carried out in China, and its international applicability is limited. Non-pharmacological treatments such as acupuncture therapy, hyperbaric oxygen therapy, repetitive transcranial magnetic stimulation, and computer-assisted cognitive rehabilitation are also being recognized as improving patient compliance (Yang et al., 2024) and interest on the basis of safety and reliability (Xu et al., 2023; Luo et al., 2024; Aprile et al., 2020). In particular, PSCI management requires collaboration between radiology, neurology, neuropsychology and rehabilitation.

## 6 Conclusion and future perspectives

In conclusion, as research into stroke and cognitive function advances, early identification of PSCI and timely intervention are of significant importance for improving patient outcomes. Neuroimaging, in particular, has emerged as a vital tool for risk stratification, yet numerous challenges remain. Current assessments are still largely confined to cross-sectional or “acute versus chronic” comparisons, failing to capture the dynamic nature of post-stroke cognitive trajectories. Future longitudinal studies must adopt multi-timepoint tracking—including baseline, 3-month, 6-month, 12-month, and 24-month follow-ups—to delineate individual patterns of recovery or decline. Such designs are essential for identifying imaging predictors of “cognitive resilience” versus “accelerated decline, “moving beyond group-level averages to enable personalized prognostic assessment. Crucially, these studies should investigate the interaction between acute stroke damage and pre-existing neurodegenerative pathology (e.g., amyloid and tau deposition) over time, as mixed pathologies increasingly underlie cognitive impairment in later life. Integrating serial multimodal imaging with fluid biomarkers will clarify how vascular injury and neurodegeneration jointly drive long-term outcomes. Ultimately, longitudinal imaging studies will facilitate dynamic risk stratification, guiding timely interventions and secondary prevention strategies tailored to individual post-stroke cognitive trajectories.
